# Design and implementation of an Internet‐Based cancer risk assessment tool: Use over 10 years

**DOI:** 10.1002/cam4.4952

**Published:** 2022-06-19

**Authors:** Michael J. LaRiviere, Ryan O'Keefe, Maribel Carpenter, Hann‐Hsiang Chao, Isabella Amaniera, Carolyn Vachani, Margaret K. Hampshire, Christina Bach, Karen Arnold‐Korzeniowski, James M. Metz, Christine Hill‐Kayser

**Affiliations:** ^1^ Department of Radiation Oncology University of Pennsylvania Philadelphia Pennsylvania USA; ^2^ Department of Internal Medicine University of Pennsylvania Philadelphia Pennsylvania USA; ^3^ Department of Radiation Oncology, Hunter Holmes McGuire Veterans Administration Medical Center Richmond Virginia USA; ^4^ Department of Radiation Oncology, Virginia Commonwealth University Richmond Virginia USA

**Keywords:** risk factors, lifestyle, surveys and questionnaires, early detection of cancer, internet, cancer risk

## Abstract

**Background:**

Prevention and early intervention can improve survival and quality of life across all cancers. Patient understanding of risk factors and associated actionable lifestyle changes and screening programs is not well understood by clinicians

**Methods:**

An Internet‐based tool, *Reduce My Risk*, was created in 2009 and made available on oncolink.org. Users voluntarily completed a survey regarding demographics and cancer risk factors, and received information about their cancer risk

**Results:**

Twenty eight thousand and one surveys were completed from 2009 to 2019. Median age was 26 years (18–101); 60% were females, 87% lived in North America, and 37% had at least a bachelor's degree. Users reported on behavioral/ modifiable risk factors: 13% were current smokers, 52% were current consumers of alcohol, and 8% of those had ≥14 drinks/week. Body mass index (BMI) was ≥30 in 19%; 74% of all surveys reported dietary risks and 36% reported infrequent exercise. Excess UV exposure was reported by 19%. Among women, 36% reported performing breast self‐examinations monthly, and 50% reported receiving clinician breast examinations at least once every 3 years. Sixty seven percent of men 55–75 years reported screening prostate specific antigen testing, with 50% receiving annual digital rectal examinations. Nonmodifiable risk factors included family cancer history (64%), genetic syndrome (3%), and cancer‐predisposing health conditions (26%)

**Conclusions:**

Ninety‐seven percent of users reported modifiable risk factors, and 60% reported ≥4 of these risk factors. Understanding detailed characteristics of a large number of respondents has the potential to improve educational interventions to reduce cancer risk through behavioral modification and cancer screening across the general public.

## Graphical abstract

An Internet‐based survey regarding demographics and cancer risk factors has generated data from 28,001 surveys completed between 2009‐2019. As 97% reported modifiable risk factors, and 60% reported ≥4 modifiable risk factors, these findings have the potential to improve educational interventions to reduce cancer risk through behavioral modification and cancer screening across the general public.

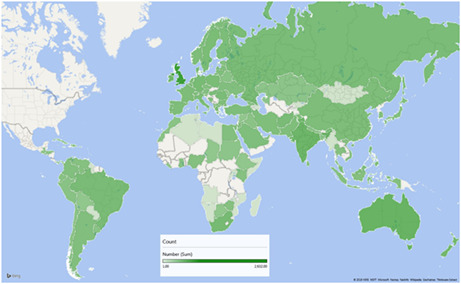

## 1 BACKGROUND

In 2003, the Institute of Medicine and National Research Council National Cancer Policy Board published a seminal report outlining the need to improve primary and secondary prevention of cancer.^1^ Primary prevention—reducing risk factors for the development of cancer—and secondary prevention—early detection of treatable cancer—have been identified as critical targets to improve survival and quality of life across a range of cancers.^1^ Risk factors for the development of cancer have been described as intrinsic, or random DNA replication errors, and non‐intrinsic risk factors, some of which are modifiable.^2^ Identifying risk factors for the development of cancer, including those that are both modifiable and non‐modifiable, has been a key public health focus over the last half‐century, supported by strengthening evidence that non‐intrinsic risk factors are responsible for as many as 60%–90% of cancers in adults.^2^ With this recognition have come meaningful improvements in screening, early detection, and overall survival. Assessing patients' cancer risk is a key task for public health researchers, primary care physicians, and oncology providers. A large body of research demonstrates associations with a wide swath of variables, including demographic factors, carcinogen exposures, family history, screening practices, and modifiable behaviors.

While public health researchers and clinicians often recommend targeted screening and interventions on the basis of these risk factors, scant literature exists studying individual patients' understanding of their own personal risk factors for cancer, and particularly the impact of co‐presence of multiple non‐modifiable and modifiable risk factors. Assessing patients' understanding is critical to developing effective, targeted behavioral interventions to reduce cancer risk.

In an effort to better inform targeted interventions for those at risk of developing cancer, we produced a publicly available, Internet‐based tool for individuals to answer a comprehensive, detailed list of questions related to their individual risk factors for cancer, with a goal of improving individual understanding of applicable modifiable and non‐modifiable risk factors. In the present report, we summarize the results of the 28,001 surveys submitted over a 10‐year period and propose future analyses and interventions that can be undertaken using these data.

## 2 METHODS

As previously described, *OncoLink* (https://www.oncolink.org) is a website housed at the Abramson Cancer Center of the University of Pennsylvania.^3^ The *OncoLink* Reduce My Risk (originally What's My Risk?) tool was created by a group of oncology physicians and nurses in 2009 as part of the *OncoLink* Risk and Prevention program (https://www.oncolink.org/risk‐and‐prevention). Beta testing and review was performed by a group of institutional and multidisciplinary specialists prior to public launch, and the program underwent a major user interface overhaul in 2018. The tool has been publicly available since 2009 on oncolink.org. Although it is only offered in English, the tool is available to users throughout the world. It is designed to provide information about individual cancer risk after users voluntarily respond to a comprehensive survey regarding demographics and cancer risk factors. Its current version can be found at https://risk.oncolink.org/. The survey was not advertised, but as the OncoLink website sees over 500,000 visitors per month, a portion of these patients discovered the tool through the OncoLink website or through searching for information about cancer risk information using a search engine. Users also learned about the tool through OncoLink cancer risk‐related web‐log (“blog”) and social media postings referencing the tool. While the survey's underlying algorithm remained unchanged over time, improvements were made to the user interface to make the content more accessible with simpler language, as well as to update content as new research became available.

Survey questions used in the tool are shown in Table S1; respondents entered numeric answers or selected from the responses shown in Tables 1, 2, 3, 4. Respondent answers are collected anonymously, with no identifiable personal data other than Internet protocol (IP) addresses, which allow identification and deletion of duplicate entries. After the survey is complete, an individualized “Risk Report” is generated to inform the user of specific cancer risks related to modifiable, non‐modifiable, and familial/ genetic risk factors identified from the user's survey. Risks are not quantified, but rather the types of cancers associated with certain risk factors (e.g., tobacco use and lung cancer) are reported. At the same time, healthy behaviors are identified and reinforced (e.g., participating in appropriate cancer screening), with education provided regarding cancer screening and risk modification, where applicable. Completing the survey provided users with the benefit of receiving educational information about reducing their personal cancer risk; compensation or other incentives were not offered.

**TABLE 1:** Responses to demographic questions

Age distribution		
<18	0%	114
18–25	44%	12,170
25–35	24%	6774
35–45	14%	4032
45–55	9%	2392
55–65	5%	1341
65–75	1%	358
75–85	0%	47
85–95	0%	5
≥95	0%	13
NA	3%	755
Gender		
Female	60%	16,878
Male	40%	11,120
Where have you spent the majority of your time		
North America (Canada, Mexico, US)	87%	24,338
Other	13%	3660
What is your race/ethnicity?		
White/Non‐Hispanic	76%	21,391
Multiple/Other	9%	2443
Asian/Pacific Islander	6%	1652
Hispanic/Latino/Latina	5%	1389
African American or Black	3%	934
Native American/Aleutian/Eskimo	1%	114
NA	0%	75
Ashkenazi Jewish Ancestry		
No	98%	27,536
Yes	2%	462
Highest educational level attained		
Some college	28%	7892
Bachelors degree	22%	6207
Graduate degree; Masters or beyond	17%	4744
Graduated high school	17%	4665
Associates degree	8%	2131
Some high school	7%	1903
Grade school	1%	397
NA	0%	59
How many people live in your household?		
1	10%	2761
2	23%	6380
3	21%	5997
4	26%	7244
5	12%	3405
6	4%	1228
7	1%	387
8+	1%	355
NA	1%	244
In which of the following settings do you live?		
Suburban area (near a city)	52%	14,418
Urban area (city)	30%	8390
Rural area (countryside, less populated areas)	18%	5147
NA	0%	43

**TABLE 2:** (A) Responses to survey questions regarding tobacco, alcohol, and other substance use. (B) Responses to survey questions regarding body mass index (BMI), diet, and exercise. (C) Responses to survey questions regarding risk factors related to sexual practices. (D) Responses to survey questions regarding risk factors related to ultraviolet light exposure. (E) Responses to occupational and environmental exposure survey questions

(A)		
Do you currently smoke cigarettes?		
No	87%	24,387
Yes	13%	3527
NA	0%	84
On average, how much do you smoke? (for those that answered yes to currently smoking)		
< 1 pack per day	74%	2602
1 pack per day	22%	761
2 packs per day	3%	102
>3 packs per day	2%	53
NA	0%	9
For how many years have you smoked? (for those that answered yes to currently smoking)		
<1 year	10%	334
1–5 years	27%	960
6–10 years	23%	799
11–20 years	23%	813
>20 years	17%	612
NA	0%	9
Did you ever smoke cigarettes?		
No	65%	18,103
Yes	23%	6501
NA	12%	3394
How much did you smoke? (for those who answered yes to did you ever smoke)		
< 1 pack per day	77%	5001
1 pack per day	19%	1240
2 packs per day	2%	159
> 3 packs per day	0%	13
NA	1%	88
For how many years did you smoke? (for those who answered yes to did you ever smoke)		
<1 year	24%	1553
1–5 years	31%	2002
6–10 years	23%	1500
11–20 years	21%	1362
NA	1%	84
How long ago did you quit? (for those who answered yes to did you ever smoke)		
<1 year ago	26%	1715
1–10 years ago	44%	2840
>10 years ago	28%	1838
NA	2%	108
Do you now or did you ever: smoke cigars or tobacco in a pipe?		
Never	90%	25,075
Former	8%	2228
Current	2%	689
NA	0%	6
Do you now or did you ever: smoke marijuana		
Never	68%	19,181
Former	22%	6141
Current	10%	2671
NA	0%	5
How much marijuana smoking do you do? (for those that answered current to do you now smoke marijuana)		
More than a few times a month	63%	1685
A few times a year to a few times a month	30%	811
I have tried it a few times in my life	6%	168
NA	0%	7
Do you now or did you ever: use oral snuff/chew/quid		
Never	94%	26,432
Former	4%	1005
Current	2%	555
NA	0%	6
Do you now or did you ever: use areca nut or betel leaf		
Never	99%	27,805
Former	1%	110
Current	0%	76
NA	0%	7
Do you now or did you ever: exotic smoking		
Never	99%	27,805
Former	1%	110
Current	0%	76
NA	0%	7
Are you exposed, on a regular basis, to significant amounts of secondhand smoke?		
No	68%	19,012
Yes, exposed regularly in the past	19%	5322
Yes, currently exposed regularly	10%	2808
NA	3%	856
Do you drink alcohol?		
Yes	52%	14,597
No	43%	12,096
I used to drink heavily, but do not anymore	5%	1304
NA	0%	4
How many drinks do you typically have, on average, in 1 week?		
0 to 3	56%	8171
4 to 7	22%	3197
8 to 10	10%	1412
11 to 13	5%	690
14 or more	8%	1096
Blank	0%	31

(B)		
Calculated BMI		
<18.5	5%	1536
18.5–24.99	50%	14,012
25–29.99	25%	7002
30–34.99	11%	3060
35–39.99	5%	1268
40–44.99	2%	596
>45	1%	399
NA	0%	128
Diet: Vegetarian/vegan?		
No	90%	25,318
Yes	10%	2680
Diet: often consume white bread, white rice, processed grains		
Yes	59%	16,504
No	41%	11,494
Diet: consume fermented, smoked, pickled foods or those preserved in salt		
No	75%	20,974
Yes	25%	7024
Diet: often consume charred/well‐done meats		
No	82%	22,857
Yes	18%	5141
Diet: consume red meat three times a week or more frequently?		
No	66%	18,591
Yes	34%	9407
How often do you engage in moderate to vigorous physical activity, above usual activities?		
<1x per week	36%	10,004
2–4x per week	48%	13,385
5 or more times per week	16%	4609
NA	0%	3
How long is your average exercise routine on any given day?		
<15 min	22%	6083
16–30 min	33%	9134
31–45 min	46%	12,782
NA	0%	2

(C)		
At what age did you first have sexual intercourse?		
Before age 18	43%	12,166
After age 18	36%	10,044
Have not had sexual intercourse	21%	5775
NA	0%	13
How many sexual partners have you had in your lifetime?		
0 to 5	65%	18,325
6 to 10	17%	4660
>10	18%	5000
Blank	0%	16
Do you or did you engage in receptive anal intercourse?		
No	84%	23,409
Yes	16%	4571
NA	0%	18
Do you or did you engage in oral sex?		
Yes	69%	19,400
No	31%	8577
NA	0%	21

(D)		
“I am a sun worshipper and love to sunbathe”		
No	89%	24,876
Yes	11%	3122
I had blistering sunburns as a child or teenager		
No	73%	20,493
Yes	27%	7505
I have more than 50 moles/birthmarks on my skin		
No	85%	23,782
Yes	15%	4216
My skin shows the signs of sun damage (lots of freckles, sun spots, etc)		
No	76%	21,178
Yes	24%	6820
Very fair (red/blond hair, fair skin)?		
No	71%	19,927
Yes	29%	8071
Have you ever used or do you currently use tanning salons/booths?		
No	80%	22,295
Yes	20%	5690
NA	0%	13
Ever diagnosed with nonmelanoma skin cx?		
No	99%	27,594
Yes	1%	356
NA	0%	48

(E)		
Have you had sustained, regular exposure to: asbestos?		
No	98%	27,438
Yes	2%	560
Have you had sustained, regular exposure to: hydrocarbons?		
No	97%	27,239
Yes	3%	759
Have you had sustained, regular exposure to: heavy metals?		
No	98%	27,416
Yes	2%	582
Have you had sustained, regular exposure to: mustard gas?		
No	100%	27,898
Yes	0%	100
Have you had sustained, regular exposure to: industrial dyes?		
No	98%	27,373
Yes	2%	625
Have you had sustained, regular exposure to: leather, rubber or wood‐working industry work		
No	98%	27,578
Yes	2%	420
Have you ever been exposed to radiation by: radiation exposure in my job above OSHA limits		
No	99%	27,703
Yes	1%	295
Have you ever been exposed to radiation by: a radiation accident? (e.g., Chernobyl)		
No	100%	27,878
Yes	0%	120
Have you ever been exposed to radiation by: at least 10 diagnostic medical tests?		
No	93%	26,155
Yes	7%	1843
Have you ever been exposed to radiation by: radiotherapy for a non‐cancer indication		
No	99%	27,756
Yes	1%	242
Have you been exposed to radon?		
Do not know, radon levels have not been tested in my home	62%	17,205
No, my house has been tested and does not contain excess radon.	37%	10,388
Yes	1%	381
Blank	0%	24

**TABLE 3:** (A) Responses to gynecologic and obstetric survey questions. (B) Responses to survey questions regarding risk‐reducing preventive care. (C) Responses to personal medical history survey questions

(A)		
At what age did you first begin menstruating?		
Before age 12	31%	5212
After age 12	65%	10,983
Unsure	4%	675
Blank	0%	8
If you gave birth, what was your age when your first child was born		
Age 30 or earlier	33%	5506
After age 30	8%	1401
Did not give birth to children	59%	9962
Blank	0%	9
Did you take hormone replacement therapy (HRT) for 2 years or longer (all ages)		
No	97%	16,308
Yes	3%	560
Blank	0%	10
Did you take hormone replacement therapy (HRT) for 2 years or longer (post‐menopausal)		
No	79%	1588
Yes	21%	419
Were you exposed (in utero or after birth) to DES (diethylstilbestrol)?		
No	99%	16,703
Yes, my mother took DES while pregnant with me	1%	141
Yes, while pregnant	0%	23
Blank	0%	11
At what age did you start menopause?		
After age 50	5%	904
Age 50 or younger	7%	1103
Not yet menopausal	88%	14,864
Did you take birth control pills at any time in your life		
Yes	67%	11,303
No	33%	5567

(B)		
Have you received the Hepatitis B vaccine?		
No	38%	10,576
Yes	62%	17,366
Blank	0%	56
Have you ever been advised to take tamoxifen or raloxifene to decrease your risk of breast cancer?		
No	99%	16,696
Yes	1%	140
How often do you perform breast self‐exam? (females only)		
I never perform breast self‐exam	33%	5580
Monthly	36%	6093
Once or twice a year	31%	5163
How often do you have a clinical breast exam by a healthcare professional? (all ages)		
At least once every 3 years	50%	8370
In the past, but not within the last 3 years	8%	1391
Never	42%	7075
How often do you have a clinical breast exam by a healthcare professional? (≥25 years)		
At least once every 3 years	70%	6449
In the past, but not within the last 3 years	11%	985
Never	19%	1708
How often do you have a clinical breast exam by a healthcare professional? (never: by age)		
<25	76%	5367
25–39	19%	1352
40–54	4%	306
55–69	1%	46
70–84	0%	3
100–114	0%	1
Have you received the Gardasil or Cervarix (HPV) vaccine? (female responses, all ages)		
No	72%	12,154
Yes	28%	4682
Have you received the Gardasil or Cervarix (HPV) vaccine? (female responses, by age)		
<18		
No	0%	47
Yes	0%	26
18–24		
No	22%	3699
Yes	21%	3594
NA	0%	24
25–34		
No	19%	3183
Yes	5%	853
NA	0%	9
35–44		
No	14%	2432
Yes	1%	85
NA	0%	7
45–54		
No	9%	1481
Yes	0%	31
NA	0%	2
55–64		
No	5%	850
Yes	0%	2
65–74		
No	1%	186
Yes	0%	3
≥75		
No	0%	24
Yes	0%	1
NA		
No	1%	249
Yes	0%	80
Have you received the Gardasil or Cervarix (HPV) vaccine? (male responses, all ages)		
No	88%	9728
Yes	12%	1381
NA	0%	13
Have you received the Gardasil or Cervarix (HPV) vaccine? (male responses, by age)		
<18		
No	0%	31
Yes	0%	10
18–24		
No	34%	3747
Yes	10%	1101
NA	0%	5
25–34		
No	23%	2558
Yes	2%	168
NA	0%	3
35–44		
No	13%	1473
Yes	0%	34
NA	0%	1
45–54		
No	8%	854
Yes	0%	20
NA	0%	1
55–64		
No	4%	473
Yes	0%	7
NA	0%	1
65–74		
No	2%	168
Yes	0%	1
≥75		
No	0%	28
Yes	0%	0
NA	0%	1
NA		
No	3%	357
Yes	0%	38
NA	0%	1
Do you have regular screening PSA test? (male respones, any age)		
No	35%	9704
Yes	5%	1403
Do you have regular screening PSA test? (by age)		
<18		
No	0%	37
Yes	0%	4
18–24		
No	41%	4607
Yes	2%	241
NA	0%	5
25–34		
No	23%	2557
Yes	2%	169
NA	0%	3
35–44		
No	12%	1335
Yes	2%	172
NA	0%	1
45–54		
No	5%	536
Yes	3%	338
NA	0%	1
55–64		
No	2%	177
Yes	3%	303
NA	0%	1
65–74		
No	0%	49
Yes	1%	120
≥75		
No	0%	21
Yes	0%	18
NA	0%	1
NA		
No	3%	387
Yes	0%	38
NA	0%	1
Do you have annual digital rectal exam? (male responses, any age)		
No	90%	9981
Yes	9%	1066
Blank	1%	73
Do you have annual digital rectal exam? (male responses, by age)		
<18		
No	0%	39
Yes	0%	2
18–24		
No	42%	4689
Yes	1%	138
NA	0%	26
25–34		
No	23%	2600
Yes	1%	109
NA	0%	20
35–44		
No	12%	1322
Yes	2%	177
NA	0%	9
45–54		
No	5%	591
Yes	3%	276
NA	0%	8
55–64		
No	2%	250
Yes	2%	226
NA	0%	5
65–74		
No	1%	73
Yes	1%	95
NA	0%	1
≥75		
No	0%	21
Yes	0%	18
NA	0%	1
NA		
No	4%	398
Yes	0%	25
NA	0%	3
Do you perform testicular self‐exam at least monthly?		
No	49%	5423
Yes	51%	5684
Blank	0%	13

(C)								
Have you ever been diagnosed with: Human Immunodeficiancy Virus (HIV)				Have you ever been diagnosed with: Gastroesophageal Reflux Disease (GERD)				
	No	100%	27,920		No	90%	25,335	
	Yes	0%	81		Yes	10%	2666	
Have you ever been diagnosed with: Human Papilloma Virus (HPV)				Have you ever been diagnosed with: Hemochromatosis				
	No	93%	26,052		No	100%	27,932	
	Yes	7%	1949		Yes	0%	69	
Have you ever been diagnosed with: Hepatitis B Virus (HBV)				Have you ever been diagnosed with: Solid organ transfer				
	No	99%	27,856		No	100%	27,946	
	Yes	1%	145		Yes	0%	55	
Have you ever been diagnosed with: Hepatitis C Virus (HCV)				Have you ever been diagnosed with: Klinefelter syndrome				
	No	100%	27,874		No	100%	27,966	
	Yes	0%	127		Yes	0%	35	
Have you ever been diagnosed with: H. pylori infection or ulcers				Have you ever been diagnosed with: Myelodysplastic syndrome (MDS)				
	No	97%	27,153		No	100%	27,960	
	Yes	3%	848		Yes	0%	41	
Have you ever been diagnosed with: Epstein Barr Virus (EBV)				Have you ever been diagnosed with: Paroxysmal nocturnal hemoglobinuria (PNH)				
	No	97%	27,271		No	100%	27,962	
	Yes	3%	730		Yes	0%	39	
Have you ever been diagnosed with: Achalasia				Have you ever been diagnosed with: Pernicious anemia				
	No	100%	27,943		No	99%	27,767	
	Yes	0%	58		Yes	1%	234	
Have you ever been diagnosed with: Atypical hyperplasia (with a breast biopsy)				Have you ever been diagnosed with: Polycystic ovary disease				
	No	99%	27,820		No	97%	27,111	
	Yes	1%	181		Yes	3%	890	
Have you ever been diagnosed with: Barrett's Esophagus				Have you ever been diagnosed with: Polycythemia Vera				
	No	99%	27,849		No	100%	27,959	
	Yes	1%	152		Yes	0%	42	
Have you ever been diagnosed with: Bulimia Nervosa (with vomiting)				Have you ever been diagnosed with: Primary sclerosing cholangitis				
	No	97%	27,267		No	100%	27,958	
	Yes	3%	734		Yes	0%	43	
Have you ever been diagnosed with: Liver disease (cirrhosis, autoimmune disease)				Have you ever been diagnosed with: Undescended testicle(s)				
	No	99%	27,847		No	100%	27,862	
	Yes	1%	154		Yes	1%	139	
Have you ever been diagnosed with: Celiac Disease				Have you ever been diagnosed with: Xeroderma pigmentosum				
	No	99%	27,756		No	100%	27,956	
	Yes	1%	245		Yes	0%	45	
Have you ever been diagnosed with: Crohn's disease				Have you or a family member ever tested positive for/been told you have: BRCA1				
	No	99%	27,820		No	99%	27,633	
	Yes	1%	181		Yes	1%	365	
Have you ever been diagnosed with: Ulcerative colitis				Have you or a family member ever tested positive for/been told you have: BRCA2				
	No	99%	27,790		No	99%	27,705	
	Yes	1%	211		Yes	1%	293	
Have you ever been diagnosed with: Colon Polyps				Have you or a family member ever tested positive for/been told you have: HNPCC				
	No	97%	27,231		No	100%	27,909	
	Yes	3%	770		Yes	0%	89	
Have you ever been diagnosed with: Down's syndrome				Have you or a family member ever tested positive for/been told you have: FAP				
	No	100%	27,963		No	100%	27,921	
	Yes	0%	38		Yes	0%	77	
Have you ever been diagnosed with: Dysplastic nevi				Have you or a family member ever tested positive for/been told you have: Other/not listed				
	No	99%	27,697		Blank	99%	27,717	
	Yes	1%	304		Yes	1%	281	
Have you ever been diagnosed with: Fanconi Anemia								
	No	100%	27,947					
	Yes	0%	54					

**TABLE 4:** (A) Reported family history of cancer by primary cancer type. (B) Reported family history of cancer by family member affected. (C) Additional family history details

(A)							
	Anal cancer	Biliary Tract Cancer (Cholangiocarcinoma)	Bladder cancer	Breast cancer	Cervical cancer	Colorectal cancer	Endometrial (Uterine) Cancer
Yes	279	86	856	6849	1053	2794	395
	*1%*	*0.3%*	*3%*	*24%*	*4%*	*10%*	*1%*
No	27,722	27,915	27,145	21,152	26,948	25,207	27,606
	*99%*	*99.7%*	*97%*	*76%*	*96%*	*90%*	*99%*
	Esophageal cancer	Gastric cancer	Head & Neck Cancer	Hodgkin's Lymphoma	Leukemia	Liver cancer (HCC)	Lung cancer (NSCLC and SCLC)
Yes	862	1005	645	438	1573	1290	5326
	*3%*	*4%*	*2%*	*2%*	*6%*	*5%*	*19%*
No	27,139	26,996	27,356	27,563	26,428	26,711	22,675
	*97%*	*96%*	*98%*	*98%*	*94%*	*95%*	*81%*
	Melanoma	Mesothelioma	Multiple Myeloma	Non‐Hodgkin Lymphoma	Non‐melanoma skin cancer	Ovarian cancer	Pancreatic cancer
Yes	2072	146	326	699	819	1453	1553
	*7%*	*1%*	*1%*	*3%*	*3%*	*5%*	*6%*
No	25,929	27,855	27,675	27,302	27,182	26,548	26,448
	*93%*	*99%*	*99%*	*98%*	*97%*	*95%*	*94%*
	Prostate cancer	Renal cell carcinoma	Sarcoma	Testicular cancer	Thyroid Cancer (all variants)	Vulvar/vaginal cancer	Brain Cancer
Yes	2627	403	348	361	682	130	1601
	*9.38%*	*1%*	*1%*	*1%*	*2%*	*0.5%*	*6%*
No	25,374	27,598	27,653	27,640	27,319	27,871	26,400
	*90.62%*	*99%*	*99%*	*99%*	*98%*	*99.5%*	*94%*

(B)					
	Father	Mother	Brother	Sister	Half sister
Yes	3782	4425	546	691	117
	*14%*	*16%*	*2%*	*2%*	*0.4%*
No	24,219	23,576	27,455	27,310	27,884
	*86%*	*84%*	*98%*	*98%*	*99.6%*
	Your mother's mother (maternal grandmother)	Your mother's father (maternal grandfather)	Your father's mother (paternal grandmother)	Your father's father (paternal grandfather)	
Yes	5395	4498	4244	3838	
	*19%*	*16%*	*15%*	*14%*	
No	22,606	23,503	23,757	24,163	
	*81%*	*84%*	*85%*	*86%*	
	Maternal aunt (mother's sister)	Maternal uncle (mother's brother)	Paternal aunt (father's sister)	Paternal uncle (father's brother)	
Yes	2610	1428	1927	1421	
	*9%*	*5%*	*7%*	*5%*	
No	25,391	26,573	26,074	26,580	
	*91%*	*95%*	*93%*	*95%*	
	Male cousin on mother's side	Female cousin on mother's side	Male cousin on father's side	Female cousin on father's side	
Yes	469	863	375	540	
	*2%*	*3%*	*1%*	*2%*	
No	27,532	27,138	27,626	27,461	
	*98%*	*97%*	*99%*	*98%*	

(C)						
At what age was this individual diagnosed?				Does the individual have metastatic disease		
	Before the age of 18		589		No	10,380
			*2%*			*37%*
	Ages 18–40		3805		Yes	7857
			*14%*			*28%*
	Ages 41–60		9789		I do not know	7918
			*35%*			*28%*
	After age 60		11,127	How many members of your family have had cancer?		
			*40%*		0	8097
	I do not know		2171			*29%*
			*8%*		1	7611
When was this individual diagnosed?						*27%*
	Within the last month		890		2	5721
			*3%*			*20%*
	Within the last year		2684		3	3128
			*10%*			*11%*
	Within the last 5 years		6095		4	1706
			*22%*			*6%*
	More than 5 years ago		13,239		5	862
			*47%*			*3%*
	I do not know		2921		6	411
			*10%*			*1%*
					7	162
						*1%*
					8	120
						*0%*
					9	30
						*0%*
					10	129
						*1%*
					NA	24
						*0%*

Italic values represent the percent of total surveys reporting the value above.

Each survey's responses are maintained in a secure database. Survey data from this convenience sample frame were extracted, and descriptive statistics were used to summarize results. Research related to these data has been approved by the University of Pennsylvania Institutional Review Board, which determined that informed consent was not required.

## 3 RESULTS

**FIGURE 1:**
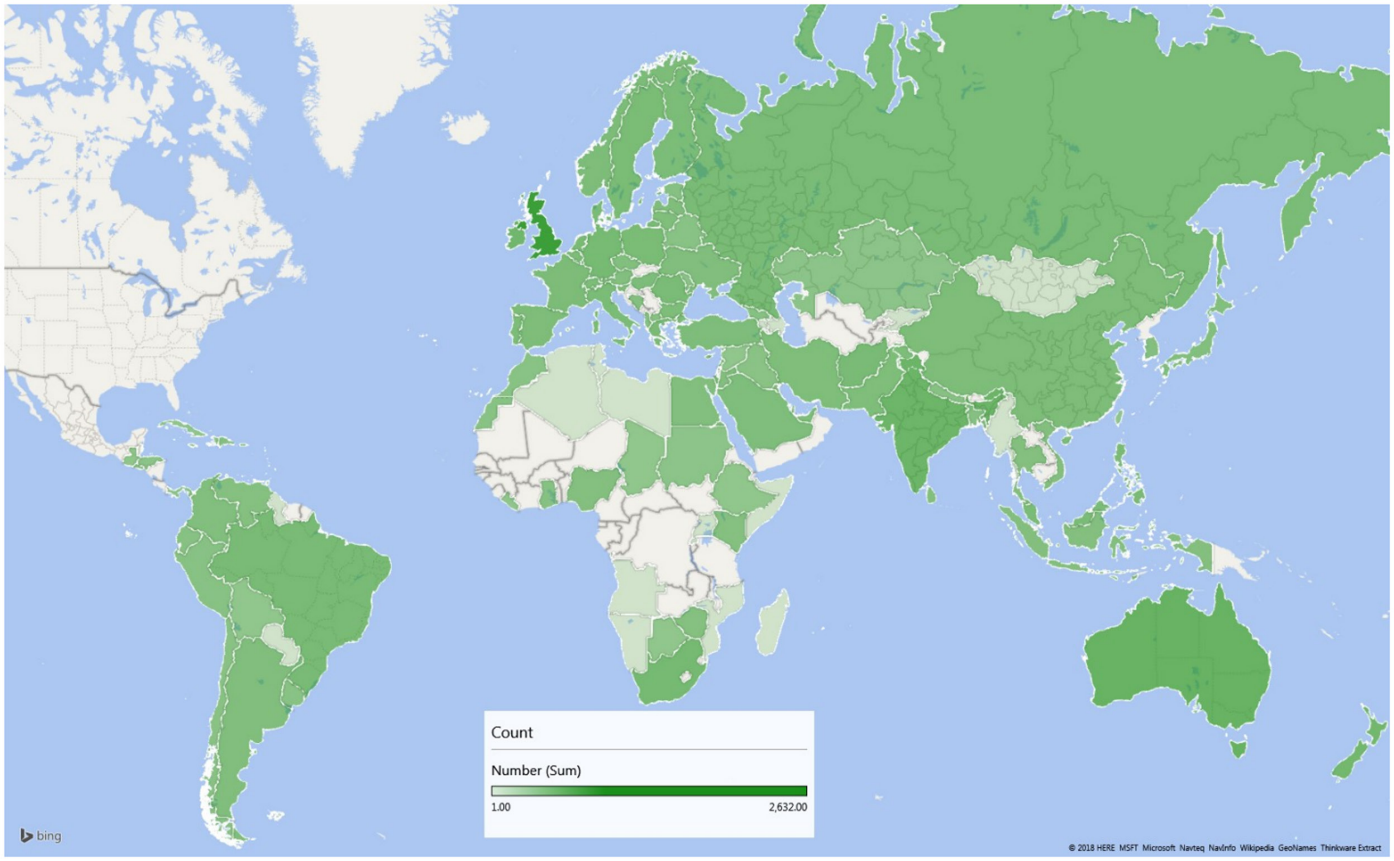
Geographic location of respondents outside of North America (“Where have you spent the majority of your life?”).

At the time of analysis, 28,001 surveys had been submitted. Sixty percent of respondents were female, and most (82%) were between 18 and 45 years old (median age 26 years, range 18–101). Seventy‐six percent identified as white/non‐Hispanic, 9% multiple/other, 6% Asian/Pacific Islander, 5% Hispanic/Latino/Latina, 3% African American or Black, and 1% Native American/Aleutian/Eskimo. Further responses to demographic questions are summarized in Table 1, and responses from all demographic groups are included in this analysis. The majority of respondents (87%) lived or spent the majority of their life in North America, followed by the United Kingdom (9%), Australia (2%), and India (1%) (Figure 1).

Modifiable risk factors with respect to smoking, alcohol, and recreational drugs are summarized in Table 2A. Thirteen percent of respondents were current smokers and 23% were former smokers. Most current and former smokers separately reported smoking one pack per day or less (96% and 97%); 60% percent of current smokers reported smoking for less than 10 years, and 26% of former smokers quit within the year prior to taking this survey. Only 10% of respondents reported cigar or pipe tobacco use and 4% oral snuff/ chew/quid. Ten percent of respondents reported current significant secondhand smoke exposure, and 19% reported prior exposure. Fifty‐two percent of individuals reported consuming alcohol, and an additional 5% reported previous heavy alcohol use. Among alcohol users, 78% reported drinking 7 or fewer drinks per week, and 8% 14 drinks or more. Sixty‐eight percent of respondents reported never smoking marijuana. Among the 10% of respondents who reported current marijuana use, the majority (63%) reported marijuana use “more than a few times” per month. A minority of respondents reported using cigar or pipe tobacco (10%), oral snuff/chew/quid (4%), areca nut or betel leaf (1%), or “exotic smoking” (1%).

To assess risk factors related to obesity, exercise, and diet, individuals first reported height and weight, from which body mass index (BMI) was calculated. Respondents then answered questions related to exercise and dietary patterns known to be associated with the development of certain cancers (Table 2B). Five percent had an underweight BMI <18.5 kg/m^2^, 50% normal (18.5–25 kg/m^2^), 25% overweight (25–30 kg/m^2^), 11% with class I obesity (30–35 kg/m^2^), 5% class II obesity (35–40 kg/m^2^), and 4% with class III obesity (≥40 kg/m^2^). Only 16% of users reported at moderate‐to‐vigorous exercise five or more times per week. In terms of diet, 34% of individuals reported eating red meat at least three times per week, and 18% consume charred or well‐done meats. Twenty‐five percent consumed fermented, smoked, pickled, or salt‐preserved foods. Fifty‐nine percent often ate white bread, white rice, or processed grains. Ten percent of respondents consumed vegetarian or vegan diets.

Sexual practices associated with certain cancer risks were also surveyed (Table 2C). Forty‐three percent of individuals reported age of first sexual intercourse before age 18; 21% reported not having had sexual intercourse. Eighteen percent of respondents reported at least 10 or more partners, 69% had engaged in oral sex, and 16% had engaged in receptive anal intercourse.

Responses regarding risk factors for the development of skin cancers are summarized in Table 2D. Of note, 1% of respondents reported a personal history of diagnosed nonmelanoma skin cancer. Twenty‐nine percent of individuals had a fair complexion, 27% reported childhood or adolescent blistering sunburns, 24% reported freckles and sun spots, and 15% had more than 50 moles or birthmarks. Modifiable risk factors included sunbathing and frequenting tanning salons (11% and 20%, respectively). The majority of respondents reported no occupational or environmental exposures (Table 2E **)**, but a small proportion of individuals reported sustained exposure to asbestos (560, 2%), hydrocarbons (759, 3%), heavy metals (582, 2%), mustard gas (100, <1%), industrial dyes (625, 2%), leather, rubber, wood‐working (420, 2%), or excess occupational radiation exposure (295, 1%). The most common source of ionizing radiation exposure was medical testing, with 1843 (7%) individuals reporting undergoing at least 10 diagnostic tests. While only 381 (1%) individuals reported known radon exposure, an additional 17,205 (62%) reported that their home had not been tested for radon.

Gynecologic and obstetric history are summarized for 16,878 women in Table 3A. Among all women, 560 (3%) had taken hormone replacement therapy for at least 2 years. Among post‐menopausal women, this proportion was 21% (419/2007), with an additional 141 women using long‐term hormone replacement therapy before menopause. One‐hundred forty‐one (1%) women reported that their mother took diethylstilbestrol (DES) while pregnant with the respondent, and 23 (<1%) women reported themselves taking DES while pregnant.

Preventive care practices surveyed are summarized in Table 3B. Sixty‐two percent of respondents reported having received the hepatitis B virus (HBV) vaccine. Among women of any age, 28% reported receipt of the human papilloma virus (HPV) vaccine; 49% of women between 18 and 25 years received the HPV vaccine. Twelve percent of all men received the HPV vaccine, in contrast to 23% of men between 18 and 25 years. Among women surveyed, 36% reported performing breast self‐examinations monthly, and 50% reported receiving a breast examination by a clinician at least once every 3 years. Among 11,120 male respondents, 51% reported performing at least monthly testicular self‐examinations. Sixty seven percent of men between 55 and 75 years reported undergoing screening prostate specific antigen testing, and 50% of men in this age group received annual digital rectal examinations. Respondents answered personal medical history questions related to 34 conditions known to be associated with the development of specific cancers (Table 3C). Gastroesophageal reflux disease (GERD) was the most commonly reported item, present among 10% of respondents, followed by a diagnosis of HPV among 7% of respondents.

Seventy‐one percent of respondents reported a family history of cancer (Table 4A–C), the majority of those reporting two or fewer family members with cancer. The most frequently reported cancers were breast cancer (24%) and lung cancer (both non‐small‐cell and small‐cell lung cancers, 19%). Nonmetastatic cancer was reported in 37% of family members, metastatic cancer in 28%, and unknown metastatic state in 28%. Maternal grandmother (19%) and grandfather (16%) were the most common family members affected, followed by mother (16%) and paternal grandmother (15%). Thirteen percent of family members were diagnosed with cancer within the year prior to the individual submitting this survey.

## 4 DISCUSSION

In the present study, we sought to understand the non‐modifiable and modifiable cancer risk factors that members of the general population harbor and self‐report in an effort to improve knowledge regarding topics for targeted education, counseling, and behavioral change. Individuals completing this comprehensive survey reported non‐modifiable risks, such as personal medical history and family history, and modifiable behaviors that may be targeted for educational and screening interventions, especially among those individuals with multiple, synergistic risk factors. Indeed, we found that not only did 97% of surveys report modifiable risk factors, but 60% of all individuals reported at least four of these risk factors. In turn, 50% of respondents reported non‐modifiable risk factors, and nearly all (98%) also reported modifiable behavioral risk factors. Taken together, these findings inform the need to identify those individuals at highest risk for the development of cancer and undertake intensive, targeted behavioral intervention.

Although this tool was freely available, demographic features of this convenience sample frame cohort differ from the general population as respondents skew younger (median age 26 years, 82% ≤45 years), female (60%), White/non‐Hispanic (76%), and better‐educated compared to the larger US and world populations. These characteristics are largely consistent with the demographics of internet surveys, which skew females, White/non‐Hispanic, and college‐educated.^4^ The majority of respondents are from North America, followed by other English‐speaking countries, likely because the tool was developed in the United States and published in English. Importantly, the generalizability of this data is limited by the fact that the majority of responses come from white, North American individuals. Future surveys should specifically seek to understand risks among non‐white populations, both within and outside of North America.

Substance use is an important modifiable risk factor for the development of multiple cancers. The majority of respondents, 64%, reported never smoking tobacco, and only 13% are current smokers. This proportion is consistent with recent estimates of smoking prevalence and also is a testament to the successful public health programs that have reduced smoking from peaks of over 50% of men and 30% of women.^5^ The fact that 26% of former smokers quit within the year prior to submitting this survey raises the question of whether those individuals were referred to the *OncoLink* Reduce My Risk tool by a clinician, or whether concern about future cancer risk led to both smoking cessation and seeking out cancer risk information. Although the majority of respondents do not use tobacco, 52% consume alcohol, a known carcinogen that is currently the target of multiple public health campaigns.^6^ Notably, the American Society of Clinical Oncology (ASCO) released a statement in 2017 underlining the increased cancer risk associated with even light alcohol consumption, additionally calling into question purported cardiovascular benefits.^7^ Among the respondents surveyed, though only 8% of those who drink alcohol would be considered heavy drinkers, over half of respondents engage in behavior that can be modified to meaningfully reduce their cancer risk. This represents an area in which patient counseling and education may have significant impact, and should be an area of focus for clinicians. Interestingly, the proportion of survey respondents who drink alcohol (52%) is actually lower than the general population (70% in the past year).^8^ This is only partially explained by the fact that 30% of respondents were under 21 years, the legal age of alcohol consumption in the United States, and that 60% of respondents were female, as women have been reported to consume alcohol at lower rates than men^9^; even analyzing the group of men ≥21 years, only 65% of respondents reported alcohol use. With respect to marijuana, although only 10% respondents reported current marijuana use, 63% of those individuals reported using marijuana at least several times per month. Among those who use marijuana, an estimated 20% consume marijuana daily,^10^ underscoring the need to understand the risks associated with heavy marijuana consumption. As the co‐presence of other cancer‐related risk factors confounds the ability to determine the cancer risk associated with marijuana specifically—(e.g., daily marijuana use is associated with tobacco use^10^) longitudinal research among individuals who consume marijuana, without concomitant tobacco use, is needed.^11^

Obesity, diet, and exercise have significant bearing on the risk of developing several cancers. Survey respondents were more likely to report non‐obese BMI compared to the general population, 82% vs. 60%.[https://www.cdc.gov/obesity/data/adult.html] Among respondents ≤19 years, 90% had non‐obese BMI, compared to 79% of 12‐19‐year‐olds in the general population.^12^ With the exception of the 59% of individuals who often consume processed carbohydrates, the majority of respondents' diets are low in foods whose preparation is associated with gastric and colorectal cancers. Despite the favorable diet and BMI profiles reported, only 16% of respondents reported moderate exercise at least 5 times per week. Thus, non‐obese individuals may represent an important group to target to recommend increased exercise to reduce the risk of certain cancers.^13^

A unique aspect of this survey is that captures both common and uncommon environmental and occupational exposures. For instance, voluntary sun and tanning salon exposures were reported among 3122 (11%) and 5690 (20%) of respondents, respectively. With respect to ionizing radiation exposure, only 37% of individuals reported that their homes have been tested for and do not contain excess radon; 381 surveys reported known radon exposure, leaving 62% of respondents with unknown potential risk with regard to radon exposure. Similarly, 1843 (7%) individuals reported receiving at least 10 diagnostic medical tests that rely on ionizing radiation. By contrast, only a very small number of surveys reported exposures to carcinogenic occupational agents, asbestos, and DES. In a similar vein, this survey captures individuals with uncommon personal medical histories, such as Fanconi anemia (54 surveys), xeroderma pigmentosum (45 surveys), primary sclerosing cholangitis (43 surveys), and others. These respondents warrant further investigation to learn more about their additional modifiable and non‐modifiable risk factors.

An additional strength of this survey is the depth of individuals' family history of cancer. In line with national trends, the most commonly reported cancers include breast (24%), lung (19%), colorectal (10%), prostate cancer (9%), and melanoma (7%). These reports appear to approximate cancer trends across recent decades (reference). Although other cancer types are overrepresented, such as brain (6%), leukemia, ovarian (5%), and cervical (4%) cancers, these in particular may be subject to inaccurate recall: Gynecologic cancers may be misclassified and brain metastases may be incorrectly considered primary brain tumors. Twenty‐eight percent of respondents, for instance, answered “I don't know” if the family member had metastatic disease.

Preventive care questions represent an interesting aspect of this survey, potentially guiding future interventions, but raise an important limitation of this survey. Namely, breast clinical and self‐examinations, breast cancer chemoprevention, prostate and testicular cancer screening, and HPV vaccination, are subject to discordant national guidelines, as well as guidelines that have changed since this survey was first made available. Additional limitations include potential self‐selection bias due to the fact that use of the survey is voluntary, and users of this survey may have a specific reason for seeking education about cancer risk reduction. For instance, 3% of respondents report that a family member was diagnosed with cancer within the last month, and 10% within the last year. A key limitation relates to the anonymous nature of this survey: Participants were not prevented from completing the survey more than once if submitting form computers with different IP addresses, potentially influencing results. Moreover, an individual's risk factors can change over time, and this is not captured by the present survey methodology.

While the purpose of the present work is to report cancer risk patterns in a large group of patients voluntarily seeking self‐education regarding cancer risk, future directions include analyses of the interactions of reported risk factors in an effort to develop targeted risk reduction interventions by clinicians and public health workers. As non‐intrinsic risk factors for the development of cancer (those that are not related to random DNA replication errors) include both non‐modifiable variables and modifiable behaviors, the results of this survey may allow for the exploration of interactions between multiple variables and behaviors, ultimately leading to better‐targeted interventions by clinicians and public health campaigns. For instance, alcohol use and cigarette smoking, which synergistically increase the risk of p16‐negative, HPV‐negative oropharynx and other aerodigestive cancers, are modifiable behaviors. Yet cigarette smoking also leads to worse outcomes for p16‐*positive*, HPV‐related oropharynx cancer, as compared to the high rates of cure now achievable for HPV‐related oropharynx cancer in non‐smokers.^14^ Prior HPV exposure is not a modifiable risk factor, but early HPV vaccination and avoidance of tobacco are behaviors that, if targeted at an early age, can meaningfully reduce future cancer risk. Other examples include sun exposure in fair‐skinned individuals, smoking among occupationally exposed workers, and diet and obesity in individuals at increased risk of breast and colon cancers. Family histories could be examined to determine whether individuals at risk for hereditary cancer syndromes are receiving appropriate screening. In a future iteration of the *OncoLink* Reduce My Risk tool, patients who take this survey upon referral from their primary care physician could consent to longitudinal follow‐up to determine the impact of risk reduction recommendations. Future work may also focus on trends in user responses over time—both before versus after the user interface overhaul, as well as, longitudinally over the 10 year reporting period. Finally, as more user responses from outside of North America are collected, data may become available for other countries' public health workers to make targeted interventions to reduce the risk of developing cancer.

Ultimately, the present findings lay the foundation for analyses that may help generate improved educational interventions to reduce cancer risk across a broad range of patients.

## AUTHOR CONTRIBUTIONS

Michael J. LaRiviere, Carolyn Vachani, Margaret K. Hampshire, Christina Bach, Karen Arnold‐Korzeniowski, James M. Metz, and Christine Hill‐Kayser conceptualized this study. Michael J. LaRiviere, Ryan O'Keefe, Maribel Carpenter, Hann‐Hsiang Chao, Isabella Amaniera, and Christine Hill‐Kayser, acquired, analyzed, and interpreted the data; and drafted this manuscript. All authors approved the final version for publication and agree to be accountable for the accuracy and integrity of all aspects of this study.

## CONFLICT OF INTEREST

All authors report no conflicts of interest.

## Supporting information


**Table S1** Survey questions.

## Data Availability

Data available on request from the authors
