# Supplementary material for: Design and implementation of an Internet‐Based cancer risk assessment tool: Use over 10 years
Source: Cancer Med. 2022 Jun 19;12(2):1744–61. doi: 10.1002/cam4.4952 (PMC9883400; doi:10.1002/cam4.4952)
Supplement: Supplementary file 1 — Table S1 Survey questions. [file CAM4-12-1744-s001.docx]

**Supplemental Table 1**

| Age |  |  |  |  |  |  |  |  |  |  |  |  |
| --- | --- | --- | --- | --- | --- | --- | --- | --- | --- | --- | --- | --- |
| What is your gender? | |  |  |  |  |  |  |  |  |  |  |  |
| Have you ever been diagnosed with cancer? (except nonmelanoma skin cancer) | | | | | | | |  |  |  |  |  |
| Where have you spent the majority of your life? | | | | |  |  |  |  |  |  |  |  |
| What is your race/ethnicity? | | |  |  |  |  |  |  |  |  |  |  |
| What is the highest educational level you obtained? | | | | |  |  |  |  |  |  |  |  |
| What is the median annual adjusted gross income of your household? | | | | | | |  |  |  |  |  |  |
| How many people live in your household? | | | |  |  |  |  |  |  |  |  |  |
| In which of the following settings do you live? | | | | |  |  |  |  |  |  |  |  |
| Do you currently smoke cigarettes? | | | |  |  |  |  |  |  |  |  |  |
| On average, how much do you smoke? | | | |  |  |  |  |  |  |  |  |  |
| For how many years have you smoked cigarettes? | | | | |  |  |  |  |  |  |  |  |
| Did you ever smoke cigarettes? | | |  |  |  |  |  |  |  |  |  |  |
| On average, how much did you smoke? | | | |  |  |  |  |  |  |  |  |  |
| For how many years did you smoke? | | | |  |  |  |  |  |  |  |  |  |
| How long ago did you quit? | | |  |  |  |  |  |  |  |  |  |  |
| Do you now or did you ever do any of the following? | | | | |  |  |  |  |  |  |  |  |
|  | smoke cigars or tobacco in a pipe | | | | use areca nut or betel leaf | | |  |  |  |  |  |
|  | smoke marijuana | |  |  | exotic smoking | |  |  |  |  |  |  |
|  | use oral snuff/chew/quid | | |  |  |  |  |  |  |  |  |  |
| How much cigar/pipe smoking do you do? | | | |  |  |  |  |  |  |  |  |  |
| How much marijuana smoking do you do? | | | |  |  |  |  |  |  |  |  |  |
| Are you exposed, on a regular basis, to significant amounts of secondhand smoke (from another | | | | | | | | |  |  |  |  |
| individual smoking cigarettes, a pipe or a cigar) at work, at home or in a regular social setting? | | | | | | | | |  |  |  |  |
| Do you drink alcohol? | |  |  |  |  |  |  |  |  |  |  |  |
| How many drinks do you typically have, on average, in one week? | | | | | |  |  |  |  |  |  |  |
| What is your height? | |  |  |  |  |  |  |  |  |  |  |  |
| What is your weight? (BMI is calculated) | | | |  |  |  |  |  |  |  |  |  |
| How would you describe your diet? | | | |  |  |  |  |  |  |  |  |  |
| How often do you engage in moderate to vigorous physical activity, above usual activities? | | | | | | | | |  |  |  |  |
| When you engage in moderate to vigorous physical activity, how long is your average exercise routine on any given day? | | | | | | | | | | |  |  |
| Which of the following describe you? | | | |  |  |  |  |  |  |  |  |  |
|  | “I am a sun worshipper and love to sunbathe” | | | | | “My skin shows the signs of sun damage (lots of freckles, sun spots, etc.)” | | | | | | |
|  | “I had blistering sunburns as a child or teenager” | | | | | “Very fair (red/blond hair, fair skin)?” | | | |  |  |  |
|  | “I have more than 50 moles/birthmarks on my skin" | | | | |  |  |  |  |  |  |  |
| Have you ever used or do you currently use tanning salons/booths? | | | | | | |  |  |  |  |  |  |
| At what age did you first have sexual intercourse? | | | | |  |  |  |  |  |  |  |  |
| How many sexual partners have you had in your lifetime? | | | | | |  |  |  |  |  |  |  |
| Do you or did you engage in receptive anal intercourse? | | | | | |  |  |  |  |  |  |  |
| Do you or did you engage in oral sex? | | | |  |  |  |  |  |  |  |  |  |
| Have you had sustained, regular exposure to any of the following? | | | | | | |  |  |  |  |  |  |
|  | asbestos |  | mustard gas | |  |  |  |  |  |  |  |  |
|  | hydrocarbons | | industrial dyes | |  |  |  |  |  |  |  |  |
|  | heavy metals | | leather, rubber or wood working industry work | | | | |  |  |  |  |  |
| Have you ever been exposed to radiation by any of the following means? (check all that apply) | | | | | | | | |  |  |  |  |
|  | radiation exposure in my job above OSHA limits | | | | | at least 10 diagnostic medical tests | | | |  |  |  |
|  | a radiation accident (e.g., Chernobyl) | | | |  | radiotherapy for a non cancer indication | | | |  |  |  |
| Have you been exposed to radon? | | | |  |  |  |  |  |  |  |  |  |
| Have you ever been diagnosed with any of the following? | | | | | |  |  |  |  |  |  |  |
|  | Human Immunodeficiency Virus (HIV) | | | |  | Down's Syndrome | |  |  |  |  |  |
|  | Human Papilloma Virus (HPV) | | |  |  | Dysplastic nevi | |  |  |  |  |  |
|  | Hepatitis B Virus (HBV) | | |  |  | Fanconi Anemia | |  |  |  |  |  |
|  | Hepatitis C Virus (HCV) | | |  |  | Gastroesophageal Reflux Disease (GERD) | | | |  |  |  |
|  | H. pylori infection or ulcers | | |  |  | Hemochromatosis | |  |  |  |  |  |
|  | Epstein Barr Virus (EBV) | | |  |  | Solid Organ Transplant | | |  |  |  |  |
|  | Achalasia |  |  |  |  | Klinefelter syndrome | |  |  |  |  |  |
|  | Atypical hyperplasia (with a breast biopsy) | | | |  | Myelodysplastic syndrome (MDS) | | | |  |  |  |
|  | Barrett's Esophagus | |  |  |  | Paroxysmal nocturnal hemoglobinuria (PNH) | | | | |  |  |
|  | Bulimia Nervosa (with vomiting) | | |  |  | Pernicious anemia | |  |  |  |  |  |
|  | Liver disease (cirrhosis, autoimmune disease) | | | | | Polycystic ovary disease | | |  |  |  |  |
|  | Celiac Disease | |  |  |  | Polycythemia Vera | |  |  |  |  |  |
|  | Crohn's Disease | |  |  |  | Primary sclerosing cholangitis | | |  |  |  |  |
|  | Ulcerative Colitis | |  |  |  | Undescended testicle(s) | | |  |  |  |  |
|  | Colon polyps | |  |  |  | Xeroderma pigmentosum | | |  |  |  |  |
| Have you or a family member ever tested positive for any of the genetic syndromes below | | | | | | | | |  |  |  |  |
| or been told you have a genetic syndrome that increases your risk of cancer? (Choose all that apply) | | | | | | | | | |  |  |  |
|  | BRCA1 | FAP |  |  |  |  |  |  |  |  |  |  |
|  | BRCA2 | Other/Not Listed | |  |  |  |  |  |  |  |  |  |
|  | HNPCC |  |  |  |  |  |  |  |  |  |  |  |
| At what age did you first begin menstruating? | | | | |  |  |  |  |  |  |  |  |
| How many children do you have? | | | |  |  |  |  |  |  |  |  |  |
| If you gave birth, what was your age when your first child was born? | | | | | | |  |  |  |  |  |  |
| Did you take hormone replacement therapy (HRT) for 2 years or longer? | | | | | | |  |  |  |  |  |  |
| Were you exposed (in utero or after birth) to DES (diethylstilbestrol)? | | | | | | |  |  |  |  |  |  |
| At what age did you start menopause? | | | |  |  |  |  |  |  |  |  |  |
| What type of cancer did your family member have? | | | | |  |  |  |  |  |  |  |  |
|  | Anal cancer | |  |  | Melanoma | |  |  |  |  |  |  |
|  | Biliary Tract Cancer (Cholangiocarcinoma) | | | | Mesothelioma | |  |  |  |  |  |  |
|  | Bladder cancer | |  |  | Multiple Myeloma | |  |  |  |  |  |  |
|  | Breast cancer | |  |  | Non-Hodgkin Lymphoma | | |  |  |  |  |  |
|  | Cervical cancer | |  |  | Non-melanoma skin cancer | | |  |  |  |  |  |
|  | Colorectal cancer | |  |  | Ovarian cancer | |  |  |  |  |  |  |
|  | Endometrial (Uterine) Cancer | | |  | Pancreatic cancer | |  |  |  |  |  |  |
|  | Esophageal cancer | |  |  | Prostate cancer | |  |  |  |  |  |  |
|  | Gastric cancer | |  |  | Renal cell carcinoma | |  |  |  |  |  |  |
|  | Head & Neck Cancer | |  |  | Sarcoma |  |  |  |  |  |  |  |
|  | Hodgkin's Lymphoma | |  |  | Testicular cancer | |  |  |  |  |  |  |
|  | Leukemia |  |  |  | Thyroid Cancer (all variants) | | |  |  |  |  |  |
|  | Liver cancer (HCC) | |  |  | Vulvar/vaginal cancer | | |  |  |  |  |  |
|  | Lung cancer (NSCLC and SCLC) | | |  | Brain Cancer | |  |  |  |  |  |  |
| Who is the family member? | | |  |  |  |  |  |  |  |  |  |  |
| At what age was this individual diagnosed? | | | |  |  |  |  |  |  |  |  |  |
| When was this individual diagnosed? | | | |  |  |  |  |  |  |  |  |  |
| Did this individual have metastatic disease (cancer that had spread to other organs)? | | | | | | | |  |  |  |  |  |
| Is the person living? | |  |  |  |  |  |  |  |  |  |  |  |
| How many members of your family have had cancer? Family members | | | | | | |  |  |  |  |  |  |
| should only include blood relatives, not those related by marriage or otherwise. | | | | | | | |  |  |  |  |  |
| Have you received the Hepatitis B vaccine? | | | |  |  |  |  |  |  |  |  |  |
| Have you ever been advised to take tamoxifen or raloxifene to decrease your risk of breast cancer? | | | | | | | | |  |  |  |  |
| How often do you perform breast self-exam? | | | | |  |  |  |  |  |  |  |  |
| How often do you have a clinical breast exam by a healthcare professional? | | | | | | |  |  |  |  |  |  |
| Do you have regular pap smears? | | | |  |  |  |  |  |  |  |  |  |
| Have you received the Gardasil or Cervarix (HPV) vaccine? | | | | | |  |  |  |  |  |  |  |
| Do you have regular screening PSA test? | | | |  |  |  |  |  |  |  |  |  |
| Do you have annual digital rectal exam? | | | |  |  |  |  |  |  |  |  |  |
| Do you perform testicular self-exam at least monthly? | | | | |  |  |  |  |  |  |  |  |
| Did you take birth control pills at any time in your life? | | | | |  |  |  |  |  |  |  |  |
